# Classical isoforms of protein kinase C (PKC) and Akt regulate the osteogenic differentiation of human dental follicle cells via both β-catenin and NF-κB

**DOI:** 10.1186/s13287-021-02313-w

**Published:** 2021-04-14

**Authors:** Oliver Pieles, Torsten E. Reichert, Christian Morsczeck

**Affiliations:** Department of Oral and Maxillofacial Surgery, University Hospital Regensburg, Franz-Josef-Strauss-Allee 11, 93053 Regensburg, Germany

**Keywords:** Dental follicle cells, Osteogenic differentiation, Mineralization, Protein kinase C, Akt, NF-κB, Canonical Wnt signaling, β-catenin

## Abstract

**Background:**

Human dental follicle cells (DFCs) are the precursor cells of the periodontium with a high potential for regenerative therapies of (alveolar) bone. However, the molecular mechanisms of osteogenic differentiation are inadequately understood. Classical isoforms of protein kinase C (PKC) are reported to inhibit osteogenesis of stem/precursor cells. This study evaluated the role of classical PKCs and potential downstream targets on the osteogenic differentiation of DFCs.

**Methods:**

DFCs were osteogenic differentiated with dexamethasone or bone morphogenetic protein 2 (BMP2). Expression of PKC and potential upstream/downstream regulators was manipulated using activators, inhibitors, and small interfering ribonucleic acid (siRNA). Expression of proteins was examined by Western blot analysis, while the activation levels of enzymes and transcription factors were examined by their phosphorylation states or by specific activation assays. Expression levels of osteogenic markers were examined by RT-qPCR (reverse transcription-quantitative polymerase chain reaction) analysis. Activity of alkaline phosphatase (ALP) and accumulation of calcium nodules by Alizarin Red staining were measured as indicators of mineralization.

**Results:**

Classical PKCs like PKCα inhibit the osteogenic differentiation of DFCs, but do not interfere with the induction of differentiation. Inhibition of classical PKCs by Gö6976 enhanced activity of Akt after osteogenic induction. Akt was also regulated during differentiation and especially disturbed BMP2-induced mineralization. The PKC/Akt axis was further shown to regulate the canonical Wnt signaling pathway and eventually nuclear expression of active β-catenin during dexamethasone-induced osteogenesis. Moreover, the nuclear factor “kappa-light-chain-enhancer” of activated B cells (NF-κB) pathway is regulated during osteogenic differentiation of DFCs and via the PKC/Akt axis and disturbs the mineralization. Upstream, parathyroid hormone-related protein (PTHrP) sustained the activity of PKC, while Wnt5a inhibited it.

**Conclusions:**

Our results demonstrate that classical PKCs like PKCα and Akt regulate the osteogenic differentiation of DFCs partly via both β-catenin and NF-κB.

**Supplementary Information:**

The online version contains supplementary material available at 10.1186/s13287-021-02313-w.

## Introduction

Dental diseases are very common and can lead to severe defects of the teeth and the periodontium, or even tooth loss. While implant-based therapies are still the gold standard, therapeutic approaches based on stem/precursor cells would be a revolutionary concept with the potential to massively improve therapeutic outcome of such diseases. Human dental follicle cells (DFCs) are the precursors of the periodontium and capable of differentiating into alveolar osteoblasts amongst several other cell types [1–5]. According to their broad differentiation potential, the molecular mechanisms underlying these processes need to be finely coordinated but are still poorly understood. The osteogenic differentiation of DFCs can be induced in vitro by treatment with either dexamethasone or bone morphogenetic protein 2 (BMP2), whereby the two differentiation protocols activate different molecular signaling cascades [6–8]. For example, the transcription factor distal-less homeobox 3 (DLX3) initiates the differentiation after BMP2 binding, while induction by dexamethasone depends on the zinc finger and BTB domain-containing protein 16 (ZBTB16) [6, 9]. Nonetheless, both differentiation pathways also overlap. For example, expression of BMP2 is upregulated in DFCs after osteogenic induction with dexamethasone [1], and parathyroid hormone-related protein (PTHrP) regulates dexamethasone-induced differentiation but also interferes with the BMP2 pathway and DLX3 [10, 11]. Furthermore, the molecular pathways during osteogenic differentiation of DFCs are presumed to be distinct from those of mesenchymal stem cells from other origins [12].

In this study, we aimed to investigate the role of protein kinase C (PKC) in the osteogenesis of DFCs and possible downstream targets. PKC is a highly conserved serine/threonine kinase and consists of at least ten different isoforms in humans with distinct functions and tissue distributions, which are involved in a variety of important molecular processes like differentiation, proliferation, apoptosis, or cell movement [13, 14]. The human PKC family can be distinguished into classical (α, βI, βI, γ), novel (δ, ε, η and θ) and atypical (ζ and ι) isoforms [14]. Previous studies showed an inhibitory effect of classical PKCs on the osteogenic differentiation of mesenchymal stem cells from non-dental origin [15, 16]. Moreover, PKCα knockout mice showed enhanced bone growth [17]. The mechanisms of action are presumably complex and consist of several downstream targets. For example, studies showed that regulation of Osterix and Msx2 by PKC is involved in the osteogenic differentiation of precursor cells [18, 19].

Besides PKC, earlier studies could already show that Akt (also called protein kinase B) sustains the osteogenic differentiation in DFCs via the expression of early growth response protein 1 (EGR1) [20]. Furthermore, both PKC and Akt are known to interact with the nuclear factor “kappa-light-chain-enhancer” of activated B cells (NF-κB) signaling pathway [21, 22], which was shown to regulate the osteogenic differentiation in dental pulp stem cells [23, 24]. Another downstream target of Akt is the canonical Wnt signaling pathway as Akt promotes osteogenesis in rat mesenchymal stem cells by phosphorylation of glycogen synthase kinase 3β (GSK3β), which regulates the activity of β-catenin [25]. The canonical Wnt signaling pathway is also involved in the osteogenic differentiation of human DFCs, albeit with an inhibitory impact [26]. Furthermore, the non-canonical Wnt signaling pathway via Wnt5a is also involved in the differentiation of DFCs [27]. We hypothesized that classical PKCs—probably together with Akt—might control osteogenic differentiation via NF-κB and Wnt signaling. This study was implemented to examine the role of classical PKC isoforms and the potential downstream mechanisms during the osteogenic differentiation of DFCs.

## Materials and methods

### Cell culture

Human DFCs were purchased from AllCells (Emeryville, USA) and grown in Dulbecco’s modified Eagle’s medium (DMEM) high glucose (Sigma-Aldrich, St. Louis, USA) supplemented with 10% fetal bovine serum (FBS, Sigma-Aldrich) and antibiotics (100 U/ml penicillin and 100 μg/ml streptomycin, Sigma-Aldrich) in 162cm^2^ or 175cm^2^ cell culture flasks (#3151 and #431080 from Corning, Corning, USA) at 37 °C and 5% CO_2_ in a humidified atmosphere. Growth medium was changed three times per week. After reaching subconfluency, DFCs were passaged by washing with phosphate-buffered saline (PBS), trypsinization, centrifugation, and resuspension in a growth medium. Concentration of cells was determined with the TC20™ Automated Cell Counter (BIO-RAD, Hercules, USA) according to the manufacturer’s instructions. For experiments or further passaging, cells were seeded with a density of 5000 cells/cm^2^ in different cell culture flasks depending on the intended experiments. Cells in passage 6–10 were used for experiments.

### Osteogenic differentiation

For experiments, osteogenic differentiation was induced in DFCs after reaching subconfluency by exchanging growth medium for an osteogenic differentiation medium (ODM) containing DMEM with 2% FBS, antibiotics, 20 mM HEPES, 10 mM β-glycerophosphate, 100 μM phospho-ascorbic acid, and 100 nM dexamethasone (all Sigma-Aldrich), or a BMP2 differentiation medium containing 50 ng/ml BMP2 (Biomol, Hamburg, Germany) instead of dexamethasone. DMEM with 2% FBS and antibiotics served as control. Differentiation medium was changed twice per week.

### PKC/Akt/NF-κB activator and inhibitor treatment

For examining the role of classical PKCs on the differentiation of DFCs, the cells were treated with 100 nM specific inhibitor Gö 6976 (Tocris) in addition to the media. To study the role of Akt, DFCs were treated with 10 μM Akt activator SC-79 (Sigma-Aldrich) or 200 nM Akt inhibitor MK-2206 (Santa Cruz Biotechnology, Dallas, USA). Besides, NF-κB was stimulated by supplementation of media with 200 nM phorbol 12-myristate 13-acetate (PMA) (Abcam, Cambridge, UK), which activates NF-κB via PKC, or inhibited with up to 500 nM (concentrations as indicated in figure legends) ACHP as general NF-κB inhibitor or CID2858522 as specific inhibitor of PKC-dependent NF-κB activation (both Tocris). All chemicals were dissolved in dimethyl sulfoxide (DMSO) and diluted in differentiation or control medium.

### Alizarin Red staining

Alizarin Red staining was performed to assess mineralization of DFCs seeded in 24-well cell culture plates (#662160 from Greiner Bio-One, Kremsmünster, Austria) after 28 days of osteogenic induction. Cells were washed with PBS, fixed with 4% formalin, and then washed three times with H_2_O. Afterwards, cells incubated in Alizarin Red solution (Merck Millipore, Billerica, USA) for 20 min, which stains accumulated calcium nodules, before another three times washing in H_2_O. Next, microscopic photographs were taken, before Alizarin Red crystals were dissolved in cetylpyridinium chloride solution (10% in PBS). Quantities of dissolved crystals were spectrophotometrically determined at OD = 595 nm. Mineralization values were normalized to the control group.

### Small interfering ribonucleic acid (siRNA) knockdown

For specific knockdown of target genes, DFCs were transfected with specific siRNAs (Qiagen, Hilden, Germany) against WNT5A (Hs_WNT5A_2), PTHLH (Hs_PTHLH_5) or PRKCA (Hs_PRKCA_5 referred to as siPRKCA #1 and Hs_PRKCA_6 referred to as siPRKCA #2) directly after seeding in 6-well cell culture plates (#353046 from Corning) using HiPerFect Transfection Reagent (Qiagen) according to the manufacturer’s instructions. Total concentration of transfected siRNAs was 5 nM. As control, cells were transfected with AllStars Negative Control siRNA (Qiagen). Three days after transfection, protein lysates were isolated and used for Western blot analysis.

### Western blot analysis

In preparation for Western blot analysis, cells that were seeded and treated in 10-cm cell culture dishes (#664160 from Greiner Bio-One), or 6-well cell culture plates if siRNA knockdown was performed before (see above), were washed with PBS, harvested in lysis buffer containing 20 mM tris HCl (pH 8.0), 137 mM NaCl, 48 mM, NaF, 1% (v/v) NP-40, 10% (v/v) glycerol, 2 mM Na_3_VO_4_, phosphatase inhibitor cocktail 3 (Sigma-Aldrich) and cOmplete™ mini protease inhibitor cocktail (Sigma-Aldrich) and centrifuged at 14,000 rpm at 4 °C for 5 min. Lysate supernatants were further used. Alternatively, cytoplasmic or nuclear protein fractions were enriched and separately isolated with the NE-PER™ Nuclear and Cytoplasmic Extraction Reagents (Thermo Scientific, Waltham, USA) according to the manufacturer’s instructions. Protein concentrations were determined with the Pierce™ BCA Protein Assay Kit (Thermo Scientific). Lysates were diluted in Laemmli sample buffer (BIO-RAD) and boiled for 5 min at 95 °C. Proteins were separated by SDS-PAGE on 4–15% Mini-PROTEAN® TXG Stain-Free™ Protein Gels (BIO-RAD), activated by UV light, and transferred onto Amersham Protran® 0.2 μm nitrocellulose membranes (Sigma-Aldrich). After taking a photograph of total membrane protein and washing in tris-buffered saline (TBS), membranes were blocked in 5% bovine serum albumin (BSA) or skimmed milk powder (according to dilution of primary antibody) in tris-buffered saline with Tween20 (TBST) for 60 min at room temperature and treated with primary antibodies against PKCα, P-PKC (corresponding to Ser660 on isoform βII), Akt, P-Akt (Ser473), P-SMAD 1/5 (Ser463/465), P-GSK3β (Ser9), NF-κB (p65), P-NF-κB (p65, Ser536), IκBα, IKKα, IKKβ (all diluted 1:1000 in BSA-containing TBST, Cell Signaling Technology, Cambridge, UK), GAPDH (diluted 1:1000 in skimmed milk powder-containing TBST, Cell Signaling Technology), Histone H3 (diluted 1:2000 in BSA-containing TBST, Cell Signaling Technology), or active β-catenin (diluted 1:750 in skimmed milk powder-containing TBST, Merck Millipore) at 4 °C overnight. After three times washing in TBST, membranes incubated in horseradish peroxidase (HRP)-linked anti-mouse IgG (1:1000 for IκBα and IKKα primary antibodies, 1:10,000 for active β-catenin primary antibody, diluted in skimmed milk powder-containing TBST, Cell Signaling Technology) or anti-rabbit IgG (1:10,000 for PKCα primary antibody, 1:1000 for all others, diluted in skimmed milk powder-containing TBST, Cell Signaling Technology) secondary antibody for 60 min at room temperature. Next, membranes were washed twice in TBST, once in PBS and once in TBS, and then a chemiluminescence signal was developed by usage of Clarity™ Western ECL substrate (BIO-RAD). Quantification of protein bands was performed densitometrically with the software Image Lab version 6.0.1 (BIO-RAD) and normalized to total lane protein and to the control group.

### NF-κB activity assay

Activity of NF-κB was determined in DFCs seeded in 10-cm cell culture dishes (#664160 from Greiner Bio-One) after 7 days of osteogenic induction. Nuclear proteins were enriched and isolated from DFCs as described in the Western blot analysis section. Activity of NF-κB subunits p50 and p65 was then determined with the TransAM® NF-κB Family Kit (Active Motif, Carlsbad, USA) according to the manufacturer’s instructions.

### RT-qPCR (reverse transcription-quantitative polymerase chain reaction) analysis

Total RNA was isolated from DFCs seeded and treated in 6-well cell culture plates (#353046 from Corning) by use of the RNeasy® Plus Mini Kit (Qiagen) according to the manufacturer’s protocol. Afterwards, the iScript™ cDNA Synthesis Kit (BIO-RAD) was used to convert mRNA into cDNA. The RT-qPCR analysis was performed with SsoAdvanced™ Universal Probes Supermix (BIO-RAD) and TaqMan probes containing primers against DLX3, RUNX2, COL1A2, and GAPDH (BIO-RAD) in a StepOnePlus Real-Time PCR System (Thermo Scientific): After 2 min activation at 95 °C, 40 cycles were conducted each consisting of 5 s denaturation at 95 °C and 30 s annealing/elongation at 60 °C. Gene expression data was normalized to the expression of the housekeeping gene GAPDH and to the control group using the delta-delta Ct method [28].

### Alkaline phosphatase (ALP) activity assay

Activity of ALP was measured in DFCs seeded in 96-well cell culture plates (#167008 from Thermo Scientific) after 7 days of osteogenic induction and NF-κB inhibitor treatment. Cells were washed twice with PBS and lysed with Triton-X solution (0.1% in PBS). ALP activity in the lysate was examined by addition of p-nitrophenylphosphate and incubation for 60 min at 37 °C. The reaction was stopped with NaOH (3 M). The quantitative conversion into yellow p-nitrophenol was spectrophotometrically measured at OD = 415 nm. ALP activity values were normalized to the control group.

### Statistical analysis

All experiments were performed in biological triplicates unless differently indicated. One-way ANOVA (analysis of variance) with Tukey’s post hoc tests and student’s t-tests were carried out with the software SPSS statistics version 25 (IBM, Armonk, USA) as indicated in the figure legends. *P* values < 0.05 were considered statistically significant and indicated in the diagrams as described in the figure legends.

## Results

### Classical PKCs inhibit the osteogenic differentiation of DFCs

**Fig. 1:**
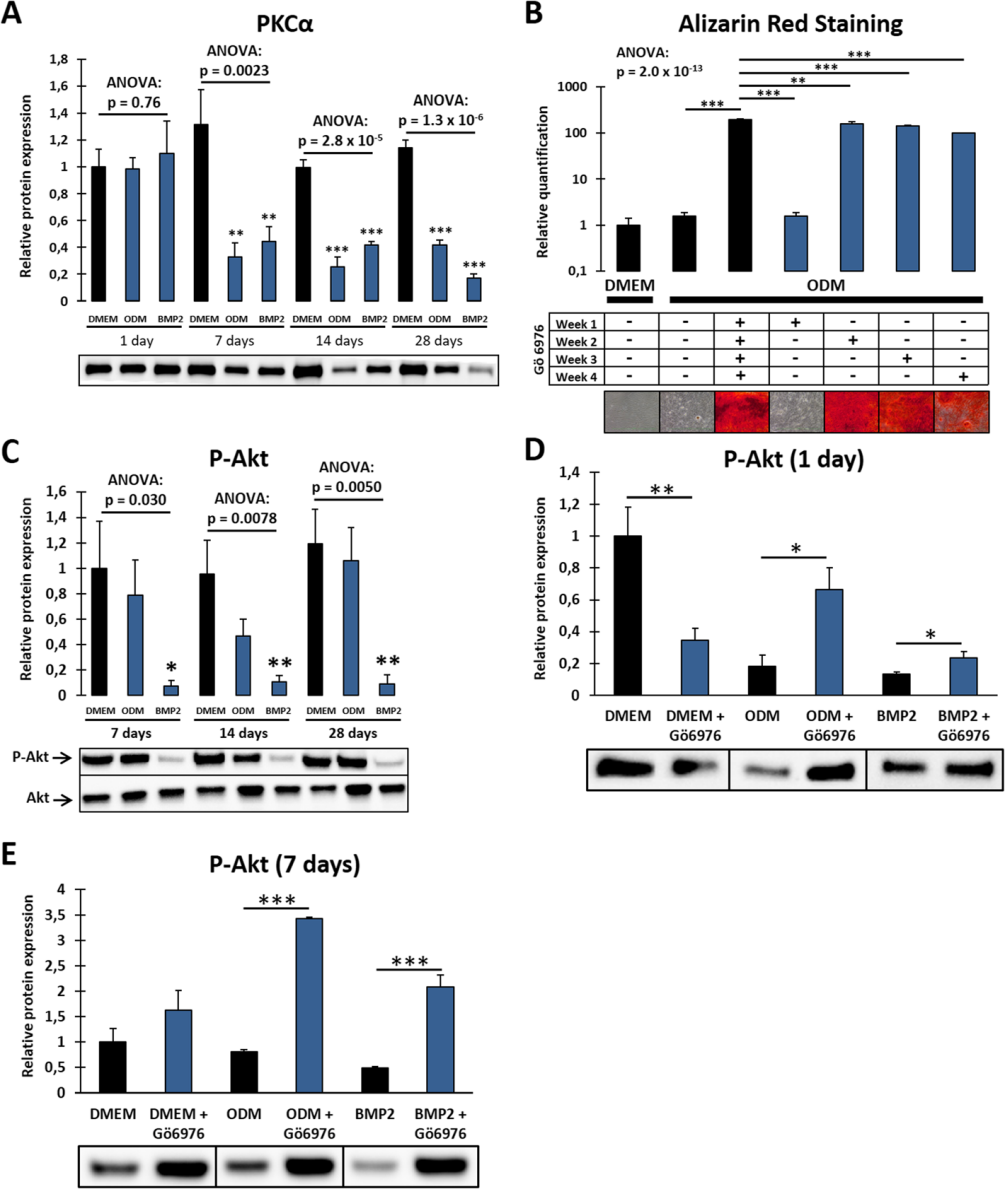
Inhibitory role of classical PKCs during osteogenic differentiation of DFCs and Akt activity during differentiation and after inhibition of classical PKCs*.* **a** Protein expression of PKCα after 1, 7, 14, or 28 days cultivation in control medium (DMEM), osteogenic differentiation medium (ODM), or BMP2 containing differentiation medium determined by Western blot analysis. **b** DFCs were cultivated for 28 days in ODM and concurrently treated with 100 nM inhibitor of classical PKCs Gö6976 for either the whole 28 days or only during the first, second, third, or fourth week of differentiation. Cells cultivated in DMEM were used as control. Mineralization of extracellular matrix was determined by Alizarin Red staining. Microscopic photographs (total width of each photograph corresponds to 1.24 mm) of stained cells are shown below the relative quantification results. **c** Protein expression of P-Akt (Ser473) in DFCs as an indicator of Akt activity after 7, 14, or 28 days cultivation in control or differentiation media as above, determined by Western blot analysis. Expression of total Akt was determined as control. **d**, **e** Protein expression of P-Akt (Ser473) in DFCs after cultivation in control or differentiation media as above for 1 (**d**) or 7 (**e**) days and simultaneous treatment with 100 nM classical PKC inhibitor Gö6976, determined by Western blot analysis. Bar charts show means + SD (*n* = 3). One-way ANOVA was performed to compare different media at the same time point including Tukey’s post hoc tests comparing the individual differentiation media with the control medium (**a**, **c**) or to compare all groups including Tukey’s post hoc tests comparing differences between continuous Gö6976 treatment in ODM and other groups in ODM (**b**). Student’s *t* test was performed to determine statistically significant differences between the control and treatment group for each medium (**d**, **e**). *p < 0.05, **p < 0.01, ***p < 0.001

Classical isoforms of PKC are known to inhibit the osteogenic differentiation of precursor cells. We first examined their expression during osteogenesis of DFCs and congruently found that classical PKCs like PKCα are also downregulated in DFCs after osteogenic induction (Fig. 1a). Furthermore, inhibition of classical PKCs strongly enhanced biomineralization of differentiating DFCs, even when applying the inhibitor for only 1 week. However, inhibition during the first week after osteogenic induction showed no effect (Fig. 1b).

### Akt activity modulates osteogenic differentiation of DFCs and is regulated by classical PKCs

**Fig. 2:**
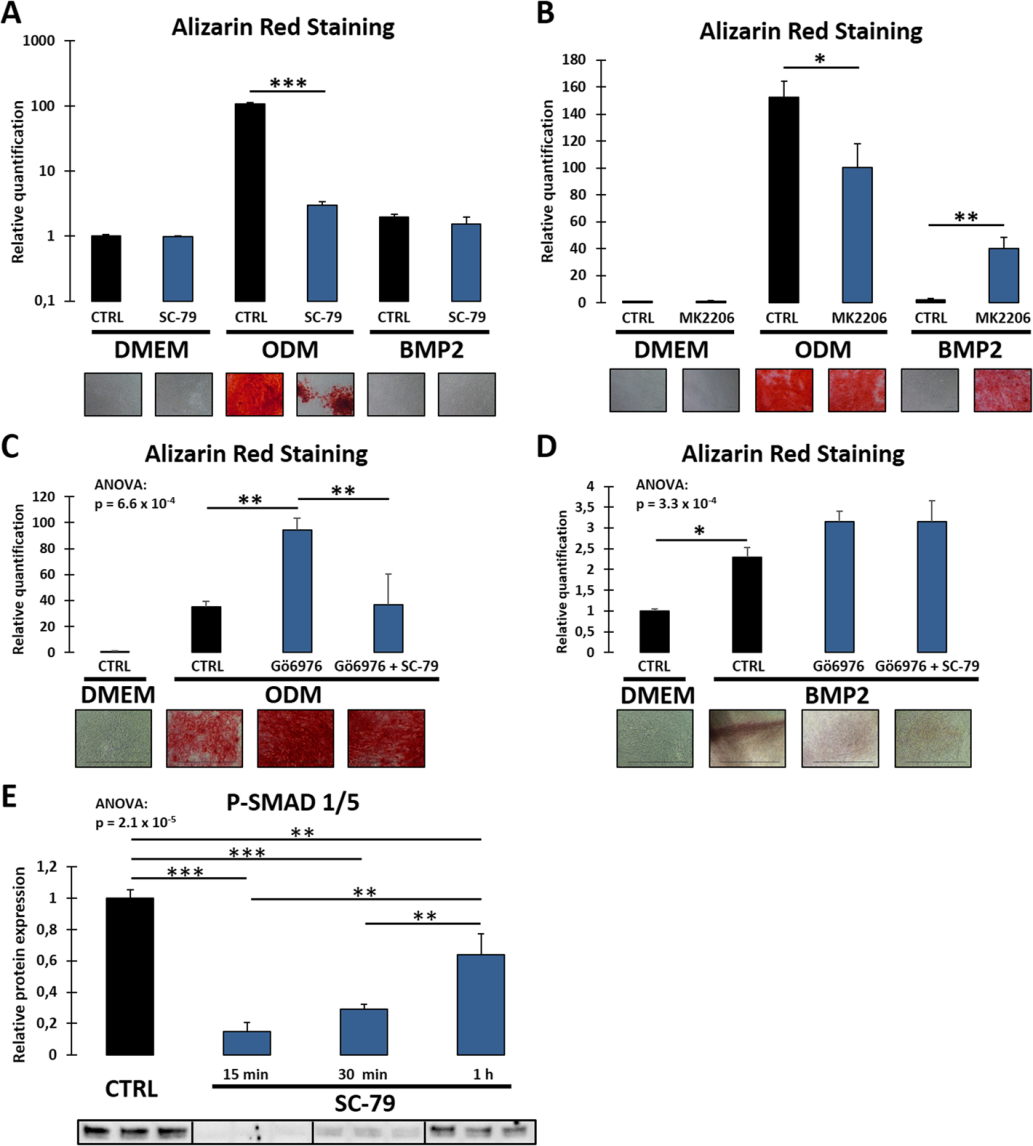
Mineralization of DFCs after treatment with Akt activators/inhibitors and interactions with PKC and BMP signaling. **a**–**d** DFCs were cultivated for 28 days in control medium (DMEM), osteogenic differentiation medium (ODM) or BMP2 containing differentiation medium, and concurrently treated with either 10 μM Akt activator SC-79 (**a**), 200 nM Akt inhibitor MK2206 (**b**), or 100 nM classical PKC inhibitor Gö 6976 alone or in combination with SC-79 (**c**, **d**). Mineralization of extracellular matrix was determined by Alizarin Red staining. Microscopic photographs (total width of each photograph corresponds to 1.24 mm) of stained cells and relative quantification results are shown. **e** DFCs were treated with 10 μM Akt activator SC-79 for 15, 30, and 60 min. Protein expression of P-SMAD 1/5 (Ser463/465) was determined by Western blot analysis. Bar charts show means + SD (*n* = 3). Student’s *t* test was performed to determine statistically significant differences between control and treatment group for each medium (**a**, **b**). One-way ANOVA was performed to compare all groups including Tukey’s post hoc tests comparing different groups in the same medium pairwise (**c**–**e**) or DMEM to ODM/BMP2 control groups (**c**, **d**). *p < 0.05, **p < 0.01, ***p < 0.001

The protein kinase Akt was considered as a potential downstream target of PKC. The expression of Akt and its Ser473-phosphorylated active form during osteogenic differentiation of DFCs were examined by Western blot analysis (Fig. 1c). We showed that Akt activity was strongly downregulated after osteogenic induction by BMP2, while only slightly—and not significantly—downregulated after dexamethasone treatment. Next, we evaluated if Akt activity was regulated after inhibition of classical PKCs for 1 and 7 days. We found that PKC inhibition strongly upregulated activity of Akt, but only after simultaneous osteogenic induction (Fig. 1d, e). Interestingly, Gö6976 treatment in control medium for 1 day downregulated Akt activity. Comparing overall results of the different media, Akt activity was equalized to a medium activity level after inhibition of classical PKCs for 1 day. Besides, expression of P-Akt was also regulated after siRNA knockdown of the gene of PKCα (Suppl. Fig. S1). We further evaluated how activation or inhibition of Akt affects the differentiation. While Akt activation by SC-79 hardly regulated the expression of osteogenic marker genes, Akt inhibition by MK2206 led to downregulation of osteogenic markers (Suppl. Fig. S2). Moreover, Akt activation strongly impaired dexamethasone-induced mineralization (Fig. 2a). However, Akt inhibition also slightly reduced mineralization after treatment with dexamethasone but could markedly enhance the mineralization capability of BMP2 treated DFCs (Fig. 2b). To evaluate the role of Akt as downstream target of PKC, we investigated if Akt stimulation interferes with the promoting impact of Gö6976 on mineralization. We found that the Akt activator abrogated significantly enhanced mineralization after PKC inhibition equal to the level without activator/inhibitor treatment in dexamethasone-treated cells (Fig. 2c), while no significant changes were observed after induction with BMP2 (Fig. 2d). Regarding the different impact of Akt on dexamethasone- versus BMP2-induced differentiation, we analyzed how Akt might interfere with BMP signaling and found that short-time activation of Akt strongly inhibited phosphorylation of SMAD 1/5 (Fig. 2e) which is a pivotal step during BMP signaling [29].

### The PKC/Akt axis modulates the canonical Wnt signaling pathway

**Fig. 3:**
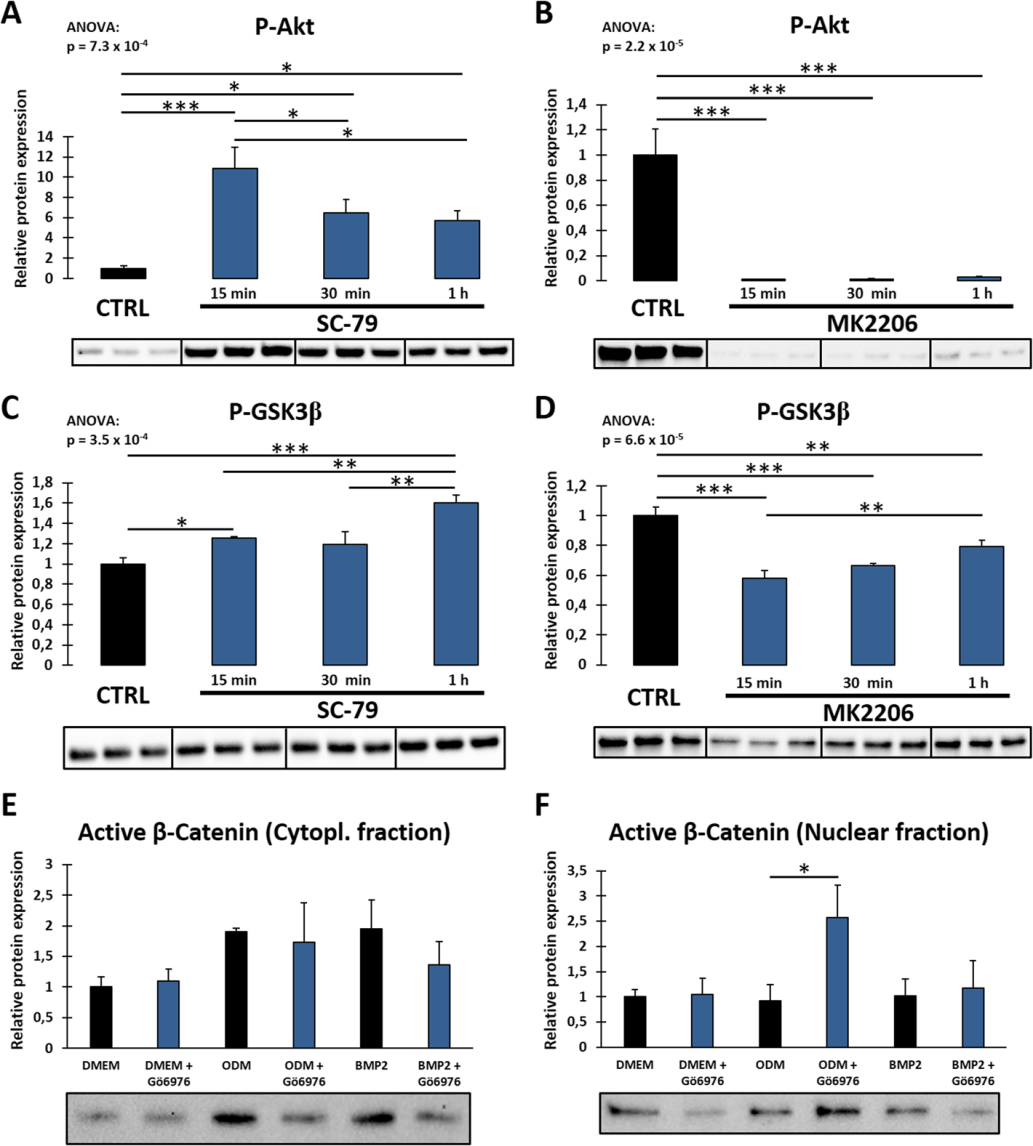
Regulation of canonical Wnt signaling pathway by PKC/Akt axis. **a**–**d** DFCs were treated with 10 μM Akt activator SC-79 (**a**, **c**) or 200 nM Akt inhibitor MK2206 (**b**, **d**) for 15, 30, and 60 min. Expression of P-Akt (Ser473) (**a**, **b**) as control and P-GSK3β (Ser9) (**c**, **d**) was determined by Western blot analysis. **e**, **f** DFCs were cultivated in control medium (DMEM), osteogenic differentiation medium (ODM), or BMP2 containing differentiation medium with and without simultaneous treatment with 100 nM classical PKC inhibitor Gö6976 for 3 days. Expression of active β-catenin was determined in Western blot analysis after enrichment of cytoplasmic (**e**) or nuclear (**f**) proteins. Bar charts show means + SD (*n* = 3). One-way ANOVA was performed to compare all groups including Tukey’s post hoc tests comparing different groups in the same medium pairwise (**a**–**d**). Student’s *t* test was performed to determine statistically significant differences between the control and treatment group for each medium (**e**, **f**). *p < 0.05, **p < 0.01, ***p < 0.001

Subsequently, we investigated if the PKC/Akt axis modulates the canonical Wnt signaling pathway. Since GSK3β can be phosphorylated at Ser9 and thus be inactivated by Akt, and—when active—modulate canonical Wnt signaling by promoting the degradation of β-catenin [25], we first examined the connection between GSK3β phosphorylation and Akt activity in DFCs (Fig. 3). Short-time stimulation with SC-79 strongly increased phosphorylation of Akt and GSK3β (Fig. 3a, c), while Akt inhibition with MK2206 distinctly decreased phosphorylation of both proteins (Fig. 3b, d). As Akt was confirmed to affect canonical Wnt signaling by inactivation of GSK3β, we next examined if classical PKCs regulate the canonical Wnt pathway. Knockdown of the gene of PKCα by specific siRNAs regulated expression of P-GSK3β (Suppl. Fig. S1). Furthermore, we analyzed subcellular localization of active β-catenin after treating DFCs with the classical PKC inhibitor Gö6976 for 3 days and isolating cytoplasmic and nuclear protein fractions separately (for control of separation see Suppl. Fig. S3). After PKC inhibition, β-catenin did not significantly change its cytoplasmic localization (Fig. 3e) but was significantly upregulated in the nucleus after osteogenic induction with dexamethasone (Fig. 3f). However, no significant differences were observed in BMP2-induced DFCs.

### The NF-κB pathway disturbs osteogenic differentiation of DFCs and is regulated by the PKC/Akt axis

**Fig. 4:**
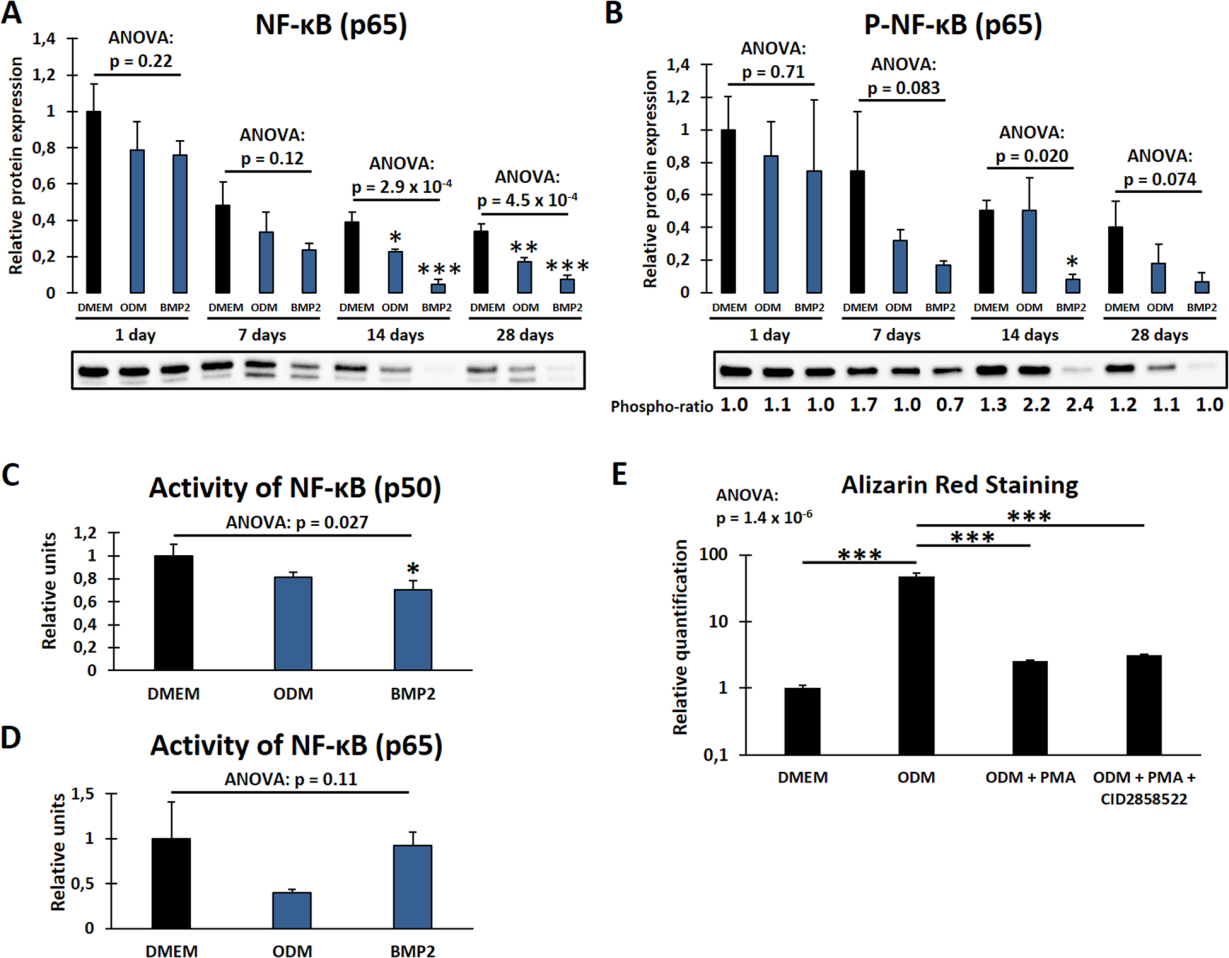
Regulation of NF-κB during osteogenic differentiation and impact of NF-κB inhibition on mineralization. **a**, **b** Expression of NF-κB (p65 subunit, **a**) and P-NF-κB (p65 subunit, Ser536, **b**) in DFCs after cultivation in control medium (DMEM), osteogenic differentiation medium (ODM) or BMP2 containing differentiation medium for 1, 7, 14 or 28 days was determined by Western blot analysis. The phospho-ratio (**b**) was calculated as ratio between phosphorylated and total NF-κB. **c**, **d** DNA binding activity of NF-κB subunits p50 (**c**) or p65 (**d**) in DFCs after 7 days cultivation in control or differentiation media as above, measured by NF-κB activity assay. **e** DFCs were cultivated in the control medium (DMEM) or osteogenic differentiation medium (ODM) and simultaneously treated with either 200 nM PKC activator PMA alone or in combination with 500 nM NF-κB inhibitor CID2858522 for 28 days. Mineralization was determined by Alizarin Red staining. Bar charts show means + SD (*n* = 3). One-way ANOVA was performed to compare different media at the same time point including Tukey’s post hoc tests comparing the individual differentiation media with the control medium (**a**–**d**) or to compare all groups including Tukey’s post hoc tests comparing the different groups in ODM pairwise or DMEM to ODM control group (**e**). *p < 0.05, **p < 0.01, ***p < 0.001

**Fig. 5:**
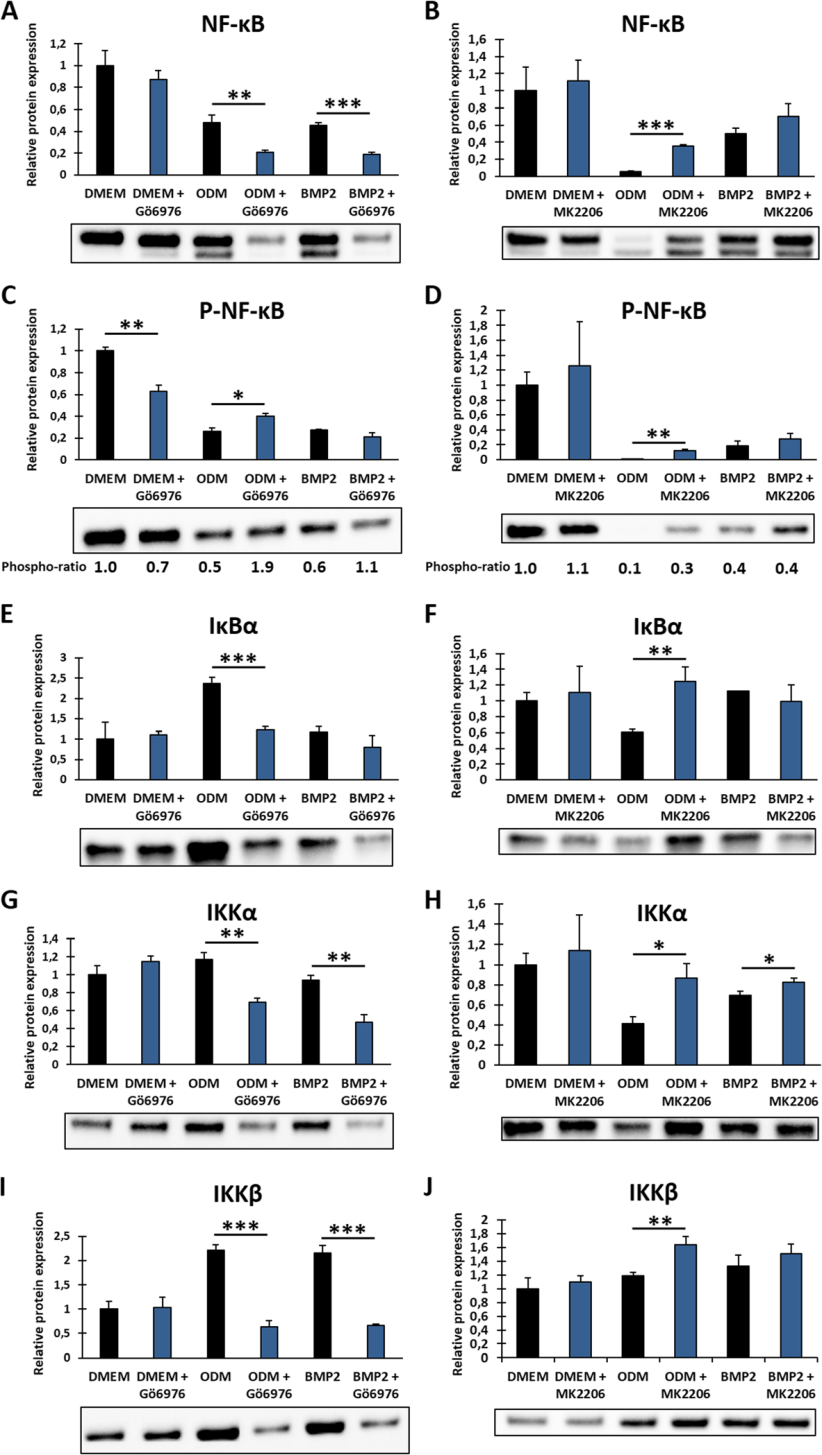
Regulation of NF-κB pathway after inhibition of PKC and Akt. DFCs were cultivated in control medium (DMEM), osteogenic differentiation medium (ODM), or BMP2 containing differentiation medium with or without simultaneous treatment with 100 nM classical PKC inhibitor Gö6976 for 7 days (**a**, **c**, **e**, **g**, **i**) or 200 nM Akt inhibitor MK2206 for 8 days (**b**, **d**, **f**, **h**, **j**). Expression of NF-κB (p65 subunit, **a**, **b**), P-NF-κB (p65 subunit, Ser536, **c**, **d**), IκBα (**e**, **f**), IKKα (**g**, **h**), and IKKβ (**I**, **J**) was determined by Western blot analysis. Phospho-ratios (**c**, **d**) were calculated as ratios between phosphorylated and total NF-κB. Bar charts show means + SD (*n* = 3). Student’s *t* test was performed to determine statistically significant differences between the control and treatment group for each medium. *p < 0.05, **p < 0.01, ***p < 0.001

As we considered NF-κB as another potential downstream target of PKC and Akt, we first analyzed how the NF-κB pathway is regulated during osteogenic differentiation of DFCs. Western blot analysis showed that the expression of NF-κB (p65 subunit) and its Ser536-phosphorylated form are downregulated after induction of differentiation, more notably in BMP2 differentiation medium (Fig. 4a, b). The ratio between phosphorylated and total NF-κB fluctuated with no distinct direction. We further evaluated the DNA binding activity of the two most abundant NF-κB isoforms p50 and p65 after osteogenic induction for 7 days and showed that their activity also decreased (Fig. 4c, d). Although downregulated during differentiation, inhibition of NF-κB by ACHP or CID2858522—which is a specific inhibitor for PKC-induced NF-κB activation—had no influence on ALP activity (Suppl. Fig. S4) and osteogenic marker gene expression (Suppl. Fig. S5), which is similar to the observations after PKC inhibition. Furthermore, only a slight and not significant increase in mineralization capability could be observed after NF-κB inhibition (Suppl. Fig. S6). Considering NF-κB as a potential downstream target of the PKC/Akt axis, we investigated if NF-κB inhibition could rescue the mineralization potential of differentiating DFCs after activation of PKC by PMA (Fig. 4e). The results showed that inhibition of PKC-induced NF-κB activity slightly enhanced mineralization after PMA treatment during differentiation, albeit this difference was not statistically significant when applying Tukey’s post hoc test after one-way ANOVA. However, reliability of this test should be considered carefully here since the variance of the *ODM* group is much larger than variance of the other groups. Thus, homoscedasticity—which is one assumption for reliability of one-way ANOVA and Tukey’s post hoc test—is violated. Hence, we additionally compared *ODM + PMA* and *ODM + PMA + CID2858522* groups separately with Student’s *t* test which stated that the difference is statistically significant (*p* = 1.1 × 10^−4^). Further experiments were performed to evaluate the regulation of NF-κB signaling after inhibition of classical PKCs and Akt during osteogenic differentiation (Fig. 5). Expression of NF-κB (p65 subunit) and its pathway-related proteins IκB, IKKα, and IKKβ decreased after PKC inhibition, but increased after inhibition of Akt. Only the expression of P-NF-κB (Ser536) was divergently regulated after PKC inhibition (Fig. 5c). However, expression of phosphorylated NF-κB was distinctly downregulated after knockdown of the gene of PKCα with at least one of two utilized siRNAs (Suppl. Fig. S1).

### Activity of PKC is sustained by PTHrP expression and inhibited by Wnt5a

**Fig. 6:**
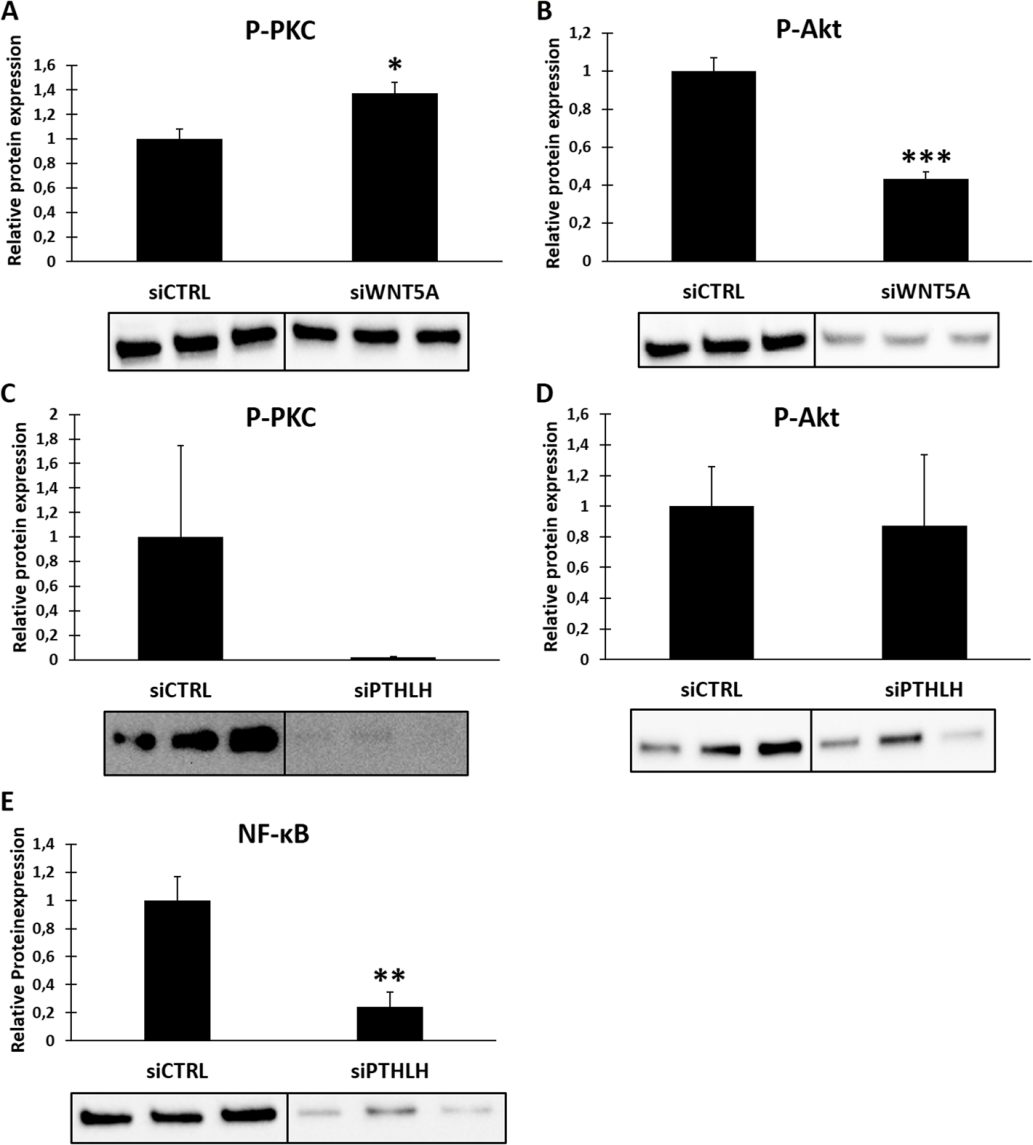
Activation of PKC and Akt by Wnt5a and PTHrP. Undifferentiated DFCs were transfected with specific siRNA against WNT5A (Wnt5a, **a**, **b**) or PTHLH (PTHrP, **c**–**e**) or control siRNA for 3 days. Expression of P-PKC (phosphorylation sites according to Ser660 on PKC βII, **a**, **c**), P-Akt (Ser473, **b**, **d**), and NF-κB (**e**) was determined by Western blot analysis. Bar charts show means + SD (*n* = 3). Student’s *t* test was performed to determine statistically significant differences in comparison to control siRNA. *p < 0.05, **p < 0.01, ***p < 0.001

Furthermore, we evaluated possible upstream regulators of the PKC/Akt signaling axis that are associated to the osteogenic differentiation of DFCs. We inhibited the expression of Wnt5a and parathyroid hormone-related protein (PTHrP) by siRNA knockdown and performed Western blots to evaluate the impact on the expression of phosphorylated PKC (at Ser660 according to PKC βII) and Akt (Ser473) as markers for the activity of the two kinases in undifferentiated DFCs (Fig. 6). The results showed that silencing of Wnt5a enhanced PKC activity, but inhibited activity of Akt (Fig. 6a, b). In contrast, PTHrP knockdown almost completely downregulated expression of phosphorylated PKC (Fig. 6c). However, inhibition of PTHrP expression did not alter Akt activity (Fig. 6d). Thus, we further investigated the influence on PTHrP inhibition on NF-κB expression as downstream target of the PKC/Akt axis and found that expression of NF-κB was also downregulated (Fig. 6e).

## Discussion

This study was conducted to investigate the role of PKC and potential downstream targets during osteogenic differentiation of DFCs. Classical PKCs like PKCα inhibit the mineralization of DFCs (Fig. 1a, b), which is consistent with previous findings in other precursor cells [16, 19]. Notably, the effect of classical PKCs on differentiation does not distinguish between osteogenic induction by dexamethasone or BMP2 and is present in mesenchymal stem cells of dental and non-dental origin. Thus, classical PKCs can be considered as key regulators in the molecular process of osteogenic differentiation. Since mineralization was not impacted when applying the specific inhibitor Gö6976 only during the first week of differentiation, but treatment during either the second, third or fourth week strongly enhanced mineralization capability of DFCs (Fig. 1b), we conclude that classical PKC isoforms regulate not the induction but the further procedure of osteogenic differentiation.

Our study showed that Akt activity in DFCs is regulated by classical PKCs (Fig. 1d and e). However, and in contrast to the results with PKC, the role of Akt is different in osteogenic differentiation induced by dexamethasone and BMP2 (Figs. 1c and 2a–d). Besides, PKC inhibition for 1 day only upregulated Akt activity in osteogenically induced DFCs, where Akt activity usually was inhibited, and not in the control cells which exhibited a higher initial expression of P-Akt (Fig. 1d). Additionally, Akt inhibition downregulated the expression of osteogenic markers (Suppl. Fig. S2). Thus, we presume that a basal Akt activity is required for the induction of osteogenic differentiation, which is in line with previous studies who found that Akt sustains the BMP2-induced osteogenic differentiation [20, 30]. However, treating DFCs with the Akt activator impaired mineralization (Fig. 2a). Furthermore, inhibition of Akt promoted mineralization after induction with BMP2 (Fig. 2b). While Akt might be indispensable for the induction of differentiation, excessive activity of the protein kinase presumably disturbs the further process. This especially affects BMP2-treated cells since Akt inhibited the phosphorylation of Smad proteins (Fig. 2e), which is required for signal transduction after binding of BMPs [29]. We presume that moderate Akt activity levels are best for successful osteogenesis and that the appropriate activity level is lower in BMP2-induced DFCs compared to dexamethasone.

The examination of potential targets of the PKC/Akt axis showed that both β-catenin and NF-κB are regulated downstream (Figs. 3 and 5). While it was already known that β-catenin, as the key molecule of the canonical Wnt signaling pathway, is involved in control of the osteogenic differentiation of DFCs [26, 31], this was new for NF-κB. In this study, PKC inhibition raised the nuclear expression of β-catenin in dexamethasone-induced DFCs (Fig. 3f). Previous studies showed that active β-catenin might promote the osteogenic differentiation of precursor cells [32, 33]. However, no influence on β-catenin localization was found in BMP2-induced cells after PKC inhibition. Since β-catenin is activated in DFCs by BMP2 signaling [26], there is probably no need for further induction of canonical Wnt signaling during BMP2-induced osteogenesis. Moreover, excessive stimulation of the canonical Wnt pathway might even disturb BMP2-induced differentiation of DFCs [34].

We showed that the NF-κB pathway is regulated by the PKC/Akt axis in DFCs and interferes with the osteogenic differentiation (Figs. 4 and 5). Downregulation of the pathway after induction with dexamethasone was not a big surprise as it was already known that glucocorticoids inhibit NF-κB [35] but since the transcription factor was also downregulated by BMP2, low levels of NF-κB might be a prerequisite for osteogenesis. This is further supported by the fact that PMA, which activates NF-κB via stimulation of PKC activity [36, 37], strongly inhibited the mineralization of DFCs. As inhibition of NF-κB after stimulation with PMA could increase mineralization capability—although only to a small extent—suppression of mineralization by PKC stimulation occurs at least partly via activation of the NF-κB pathway (Fig. 4e). This is in line with the results of other studies who showed that activation of NF-κB attenuates the osteogenic differentiation of precursor cells [38–40]. Activity of PKC is further related to the process of tooth eruption by stimulating the expression of tumor necrosis factor α (TNF-α) [41], which results in the activation of NF-κB [42]. Several studies found that the NF-κB pathway promotes osteoclastogenesis [43–45]. During tooth development, inhibition of classical PKCs and NF-κB might shift the cell functionality from tooth eruption to osteogenesis.

Our results further showed that PKC activation highly depends on PTHrP expression (Fig. 6c - e). This is in line with earlier studies that exhibited an inhibitory effect of PTHrP on osteogenic differentiation markers in DFCs [10, 46]. However, another study revealed a supporting effect of nuclear localized PTHrP on osteogenesis of DFCs [11]. We assume that the role of PTHrP as a regulator of osteogenic differentiation in DFCs is versatile and that PKC is only one of several targets. On the contrary, the upstream mediator Wnt5a was shown to inhibit PKC activity in DFCs (Fig. 6a), which subsequently affects Akt activity (Fig. 6b). This assumes a promoting role of Wnt5a for the osteogenic differentiation, which is in line with observations of previous studies [27, 47, 48]. It is also known that Wnt5a supports the viability of DFCs [27]. Since both dexamethasone and BMP2 were already shown to increase proliferation in several cell types [49–52], regulation of cell viability might affect osteogenic differentiation. Furthermore, numerous studies demonstrated that PKC and Akt influence cell proliferation [53–57]. Moreover, PKC is recently considered to be an important tumor suppressor [58]. Besides the investigated signaling pathways in this study, regulation of cell viability is possibly another important mechanism how classical PKCs might regulate the osteogenic differentiation of DFCs, which needs to be further evaluated in the future.

One major limitation of this study is that the explored signaling pathways explain the regulatory role of the PKC/Akt axis only partly and more studies are required to fully understand the role of the protein kinases during differentiation of precursor cells. Moreover, gene expression of osteogenic markers was hardly altered or even downregulated after induction especially with dexamethasone, which is in line with earlier observations [6], and after Akt activation or inhibition (Suppl. Fig. S2). We presume that basal expression levels of osteogenic markers are sufficient for differentiation and further upregulation is not required, which makes it harder to explore if manipulation of molecular signaling pathways influences osteogenesis. Notably, mineralization was stronger after osteogenic induction with dexamethasone compared to BMP2 (Fig. 2a, b). Hence, it is intriguing that a reliable mineralization of BMP2-induced DFCs could be achieved by inhibition of Akt, whereas dexamethasone-induced differentiation depends on other molecular pathways and especially benefits from inhibition of classical PKCs. Prospectively, a fundamental understanding of how PKCs and Akt modulate osteogenic differentiation of DFCs will be an important step in developing new stem cell-based therapies for dental diseases and tooth loss.

## Conclusion

**Fig. 7:**
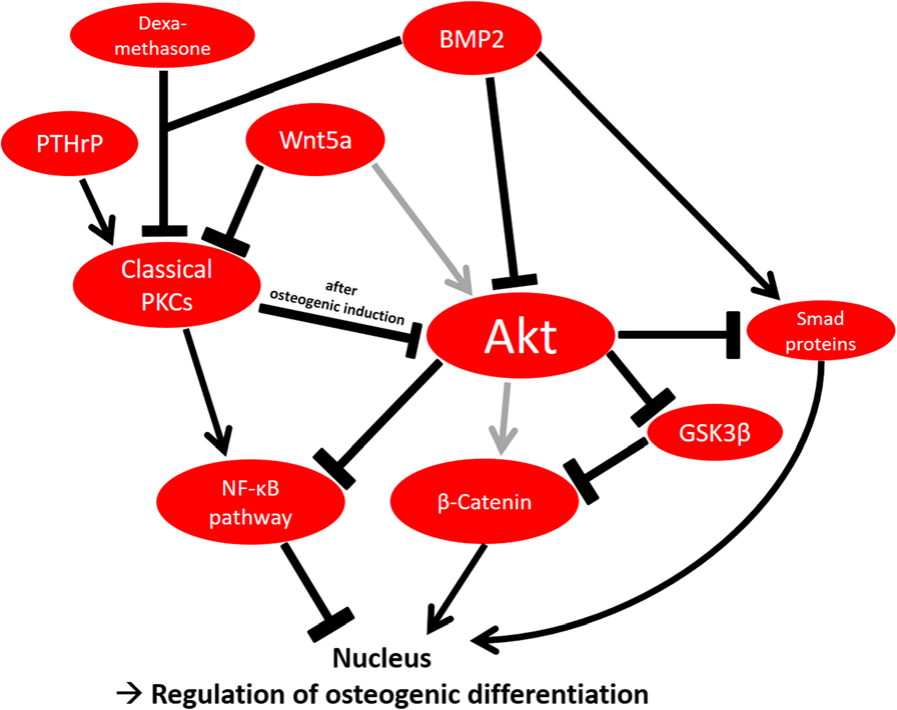
Graphical summary of the investigated signaling pathways during osteogenic differentiation of DFCs. The osteogenic differentiation in DFCs can be induced by dexamethasone or BMP2. Induction by dexamethasone leads to downregulation of classical PKCs, while induction by BMP2 inhibits both classical PKCs and Akt. Activity of classical PKCs is sustained by PTHrP and can be inhibited by Wnt5a. In osteogenic differentiating cells, classical PKCs inhibit the activity of Akt, while Akt was shown to inhibit the NF-κB pathway and sustain the activity of β-catenin by phosphorylation and thereby inhibition of GSK3β. Classical PKCs stimulate the NF-κB pathway. Eventually, the transcription factors NF-κB and β-catenin modulate the osteogenic differentiation of DFCs. Furthermore, the BMP2/Smad signaling pathway, which is pivotal for BMP2-induced osteogenic induction, is disturbed by Akt

Classical PKCs regulate the activity of Akt in DFCs. Both protein kinases regulate the osteogenic differentiation and mineralization of DFCs—at least partly—via the downstream targets β-catenin and NF-κB. While dexamethasone-induced differentiation is notably enhanced after inhibition of classical PKCs, BMP2-induced osteogenesis can be improved especially by Akt inhibition, because Akt disturbs the BMP2/Smad signaling pathway. The activity of PKC is dependent on PTHrP expression and can be regulated by Wnt5a. Figure 7 graphically summarizes the findings of our study.

## Supplementary Information

**Additional file 1: Figure S1.** *Regulation of downstream targets after siRNA knockdown of PRKCA (PKCα).* Undifferentiated DFCs were transfected with two specific siRNAs against PRKCA (PKCα) or control siRNA for three days. Expression of PKCα, P-Akt (Ser473), P-GSK3β (Ser9), P-NF-κB (p65 subunit, Ser536) and GAPDH was determined by Western blot analysis (*n* = 1).

**Additional file 2: Figure S2**. *Gene expression of osteogenic marker genes in DFCs after treatment with Akt activators/inhibitors.* DFCs were cultivated for seven days in control medium (DMEM), osteogenic differentiation medium (ODM) or BMP2 containing medium, and concurrently treated with either 10 μM Akt activator SC-79 (**A**, **B**, **E**) or 200 nM of Akt inhibitor MK2206 (**B**, **D**, **F**). Relative expression of the genes DLX3 (**A**, **B**), RUNX2 (**C**, **D**) and COL1A2 (**E**, **F**) was determined by RT-qPCR. Bar charts show means + SD (*n* = 3). Student’s *t*-test was performed to determine statistically significant differences in compare to the control in the same medium. *p < 0.05, **p < 0.01. *DLX3* Distal-less homeobox 3, *RUNX2* Runt-related transcription factor 2, *COL1A2* Collagen type I alpha 2 chain.

**Additional file 3: Figure S3**. *Enrichment of cytoplasmic and nuclear fractions in DFCs.* Western blots of GAPDH (control for cytoplasmic enrichment) and Histone H3 (control for nuclear enrichment) show separation of cytoplasmic and nuclear proteins. Total lane protein is shown below. The samples were used for the Western blots in Fig. 3e and f. *GAPDH* Glyceraldehyde 3-phosphate dehydrogenase.

**Additional file 4: Figure S4**. *ALP activity after NF-κB inhibition.* DFCs were cultivated in osteogenic differentiation medium (ODM, **A**, **C**) or BMP2 containing differentiation medium (**B**, **D**) and simultaneously treated with different concentrations of NF-κB inhibitors ACHP (**A**, **B**) or CID2858522 (**C**, **D**) or cultivated in control medium (DMEM) for 7 days before activity of ALP (alkaline phosphatase) was measured. Bar charts show means + SD (*n* = 3). One-way ANOVA was performed to compare all groups including Tukey’s post hoc tests comparing different groups in the same medium pairwise or DMEM to ODM/BMP2 control group. *p < 0.05, **p < 0.01.

**Additional file 5: Figure S5**. *Osteogenic marker gene expression after NF-κB inhibition.* DFCs were cultivated in osteogenic differentiation medium (ODM) and simultaneously treated with 500 nM NF-κB inhibitor ACHP for 3 days. Gene expression of DLX3 (**A**), RUNX2 (**B**) and COL1A2 (**C**) was measured by RT-qPCRs. Bar charts show means + SD (*n* = 3). *DLX3* Distal-less homeobox 3, *RUNX2* Runt-related transcription factor 2, *COL1A2* Collagen type I alpha 2 chain. Student’s *t*-test was performed to compare treatment and control group, but no significant difference was detected.

**Additional file 6: Figure S6**. *Mineralization after NF-κB inhibition.* DFCs were cultivated in osteogenic differentiation medium (ODM, **A**, **C**) or BMP2 containing differentiation medium (**B**, **D**) and simultaneously treated with different concentrations of NF-κB inhibitors ACHP (**A**, **B**) or CID2858522 (**C**, **D**) or cultivated in control medium (DMEM) for 28 days before mineralization was determined by Alizarin Red staining (total width of each photograph corresponds to 1.24 mm). Bar charts show means + SD (*n* = 3). One-way ANOVA was performed to compare all groups including Tukey’s post hoc tests comparing different groups in the same medium pairwise or DMEM to ODM/BMP2 control group. *p < 0.05, **p < 0.01, ***p < 0.001.

## Data Availability

The raw data is available from the authors on reasonable request.

## Abbreviations

ALP: Alkaline phosphatase
ANOVA: Analysis of variance
BMP2: Bone morphogenetic protein 2
BSA: Bovine serum albumin
DFC: Dental follicle cells
DLX3: Distal-less homeobox 3
DMEM: Dulbecco’s modified Eagle’s medium
DMSO: Dimethyl sulfoxide
EGR1: Early growth response protein 1
FBS: Fetal bovine serum
GSK3β: Glycogen synthase kinase 3β
HRP: Horseradish peroxidase
NF-κB: Nuclear factor “kappa-light-chain-enhancer” of activated B cells
ODM: Osteogenic differentiation medium
PBS: Phosphate-buffered saline
PKC: Protein kinase C
PMA: Phorbol 12-myristate 13-acetate
PTHrP: Parathyroid hormone-related protein
RT-qPCR: Reverse transcription-quantitative polymerase chain reaction
siRNA: Small interfering ribonucleic acid
TBS: Tris-buffered saline
TBST: Tris-buffered saline with Tween20
TNF-α: Tumor necrosis factor α
ZBTB16: Zinc finger and BTB domain-containing protein 16
