# Supplementary figures and images for: Classical isoforms of protein kinase C (PKC) and Akt regulate the osteogenic differentiation of human dental follicle cells via both β-catenin and NF-κB
Source: Stem Cell Res Ther. 2021 Apr 14;12:242. doi: 10.1186/s13287-021-02313-w (PMC8048169; doi:10.1186/s13287-021-02313-w)

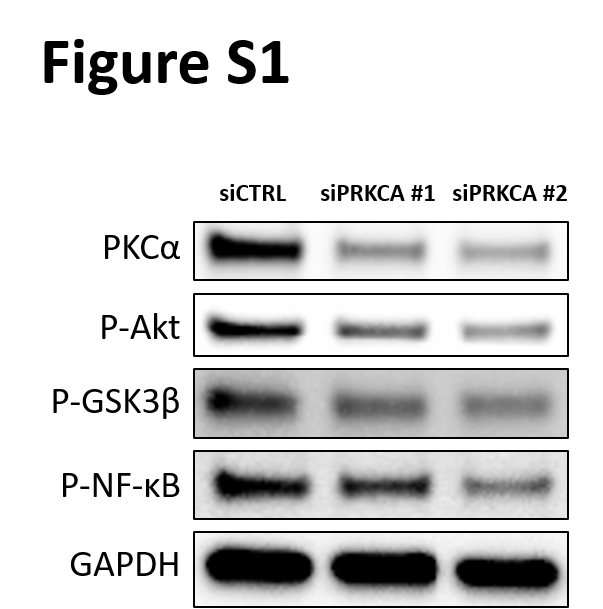

Supplement: Supplementary file 1 — Additional file 1: Figure S1. Regulation of downstream targets after siRNA knockdown of PRKCA (PKCα). Undifferentiated DFCs were transfected with two specific siRNAs against PRKCA (PKCα) or control siRNA for three days. Expression of PKCα, P-Akt (Ser473), P-GSK3β (Ser9), P-NF-κB (p65 subunit, Ser536) and GAPDH was determined by Western blot analysis (n = 1). [file 13287_2021_2313_MOESM1_ESM.tif]

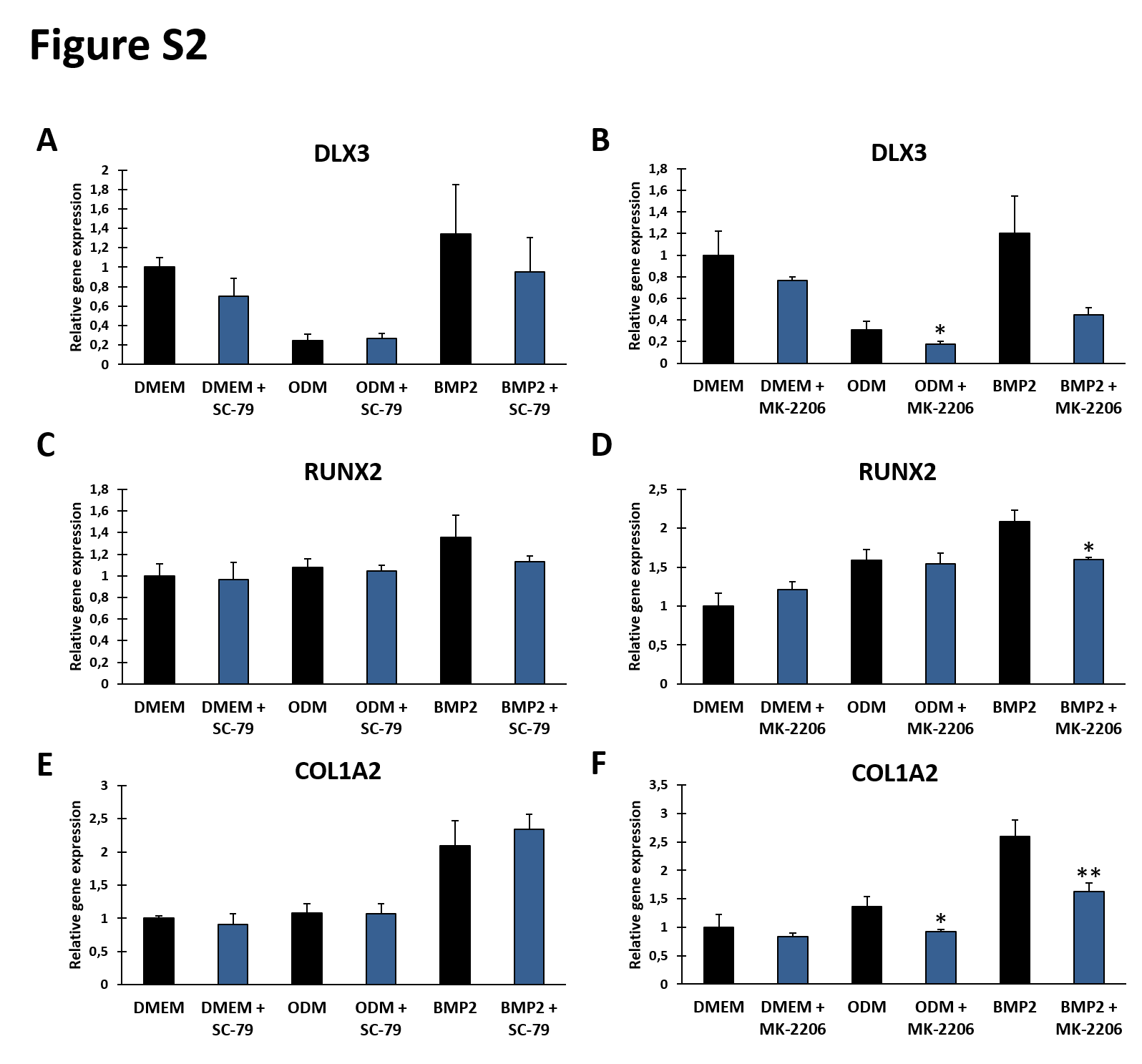

Supplement: Supplementary file 2 — Additional file 2: Figure S2. Gene expression of osteogenic marker genes in DFCs after treatment with Akt activators/inhibitors. DFCs were cultivated for seven days in control medium (DMEM), osteogenic differentiation medium (ODM) or BMP2 containing medium, and concurrently treated with either 10 μM Akt activator SC-79 (A, B, E) or 200 nM of Akt inhibitor MK2206 (B, D, F). Relative expression of the genes DLX3 (A, B), RUNX2 (C, D) and COL1A2 (E, F) was determined by RT-qPCR. Bar charts show means + SD (n = 3). Student’s t-test was performed to determine statistically significant differences in compare to the control in the same medium. *p < 0.05, **p < 0.01. DLX3 Distal-less homeobox 3, RUNX2 Runt-related transcription factor 2, COL1A2 Collagen type I alpha 2 chain. [file 13287_2021_2313_MOESM2_ESM.tif]

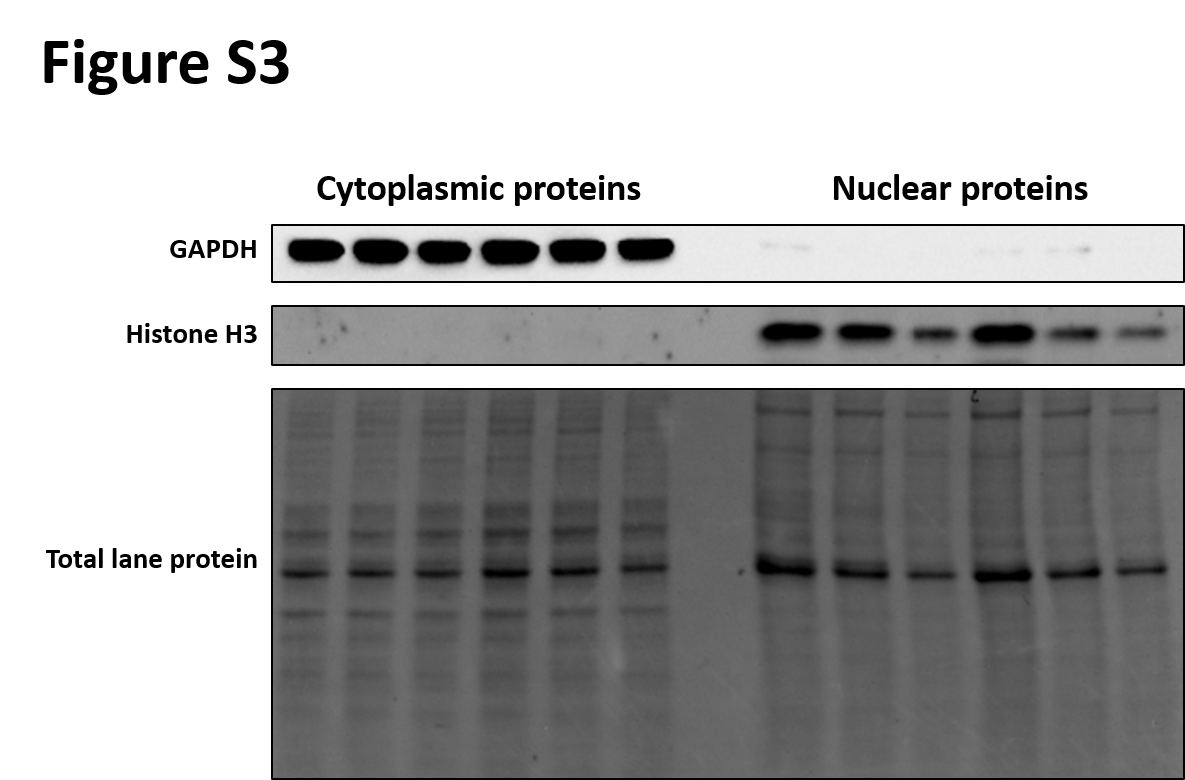

Supplement: Supplementary file 3 — Additional file 3: Figure S3. Enrichment of cytoplasmic and nuclear fractions in DFCs. Western blots of GAPDH (control for cytoplasmic enrichment) and Histone H3 (control for nuclear enrichment) show separation of cytoplasmic and nuclear proteins. Total lane protein is shown below. The samples were used for the Western blots in Fig. 3e and f. GAPDH Glyceraldehyde 3-phosphate dehydrogenase. [file 13287_2021_2313_MOESM3_ESM.tif]

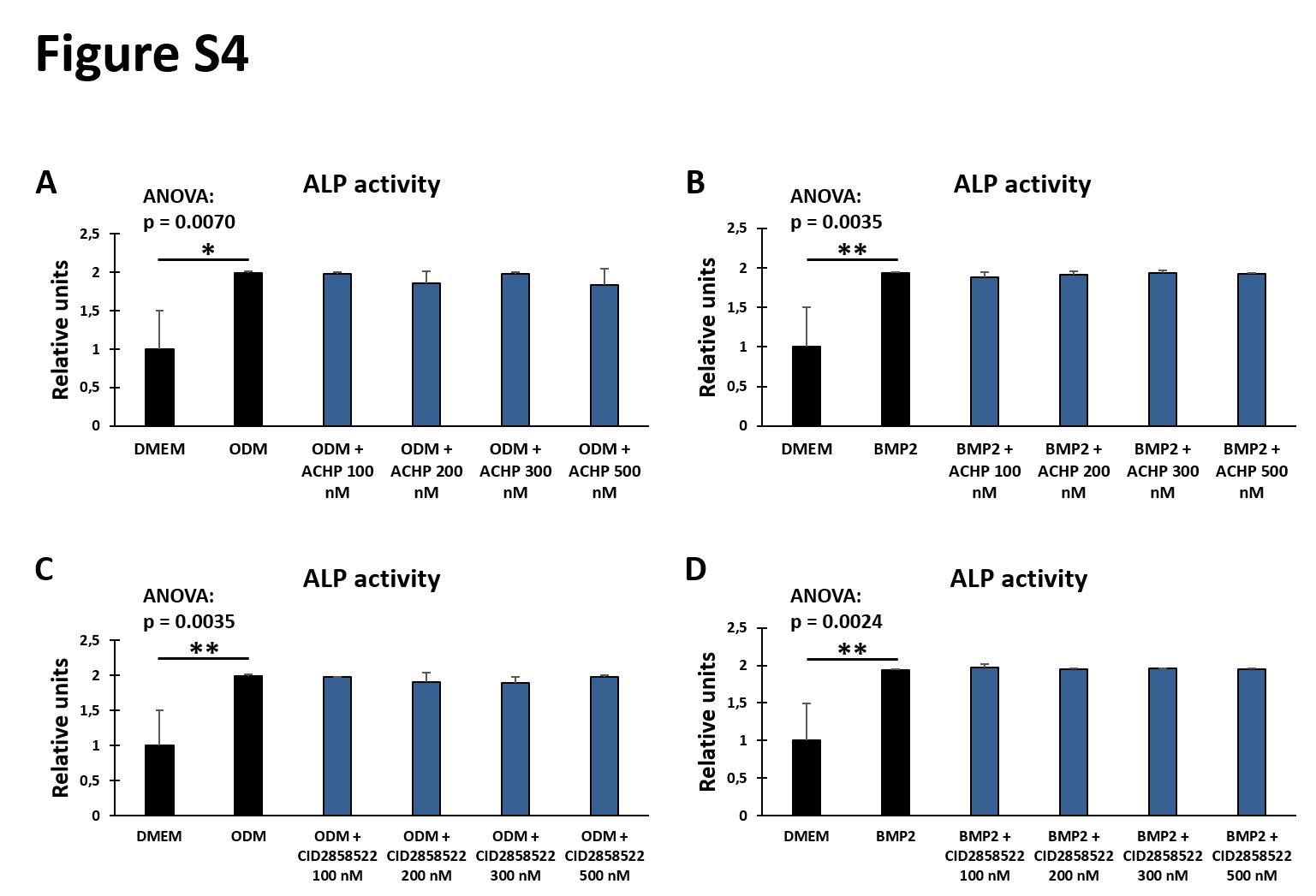

Supplement: Supplementary file 4 — Additional file 4: Figure S4. ALP activity after NF-κB inhibition. DFCs were cultivated in osteogenic differentiation medium (ODM, A, C) or BMP2 containing differentiation medium (B, D) and simultaneously treated with different concentrations of NF-κB inhibitors ACHP (A, B) or CID2858522 (C, D) or cultivated in control medium (DMEM) for 7 days before activity of ALP (alkaline phosphatase) was measured. Bar charts show means + SD (n = 3). One-way ANOVA was performed to compare all groups including Tukey’s post hoc tests comparing different groups in the same medium pairwise or DMEM to ODM/BMP2 control group. *p < 0.05, **p < 0.01. [file 13287_2021_2313_MOESM4_ESM.tif]

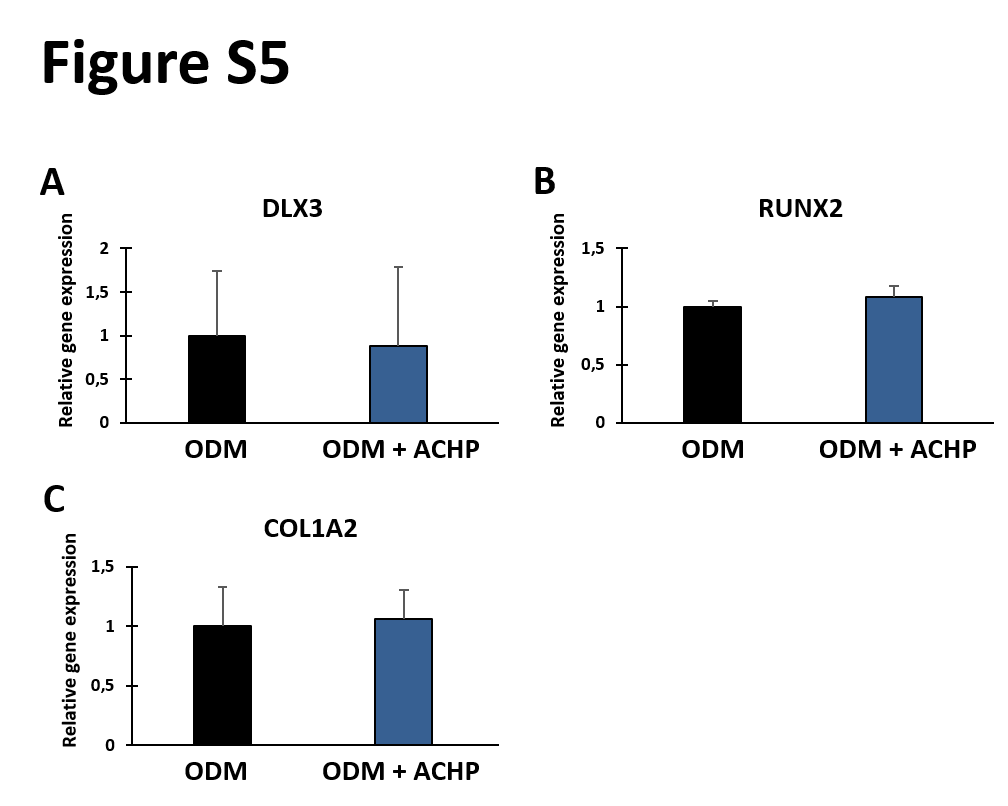

Supplement: Supplementary file 5 — Additional file 5: Figure S5. Osteogenic marker gene expression after NF-κB inhibition. DFCs were cultivated in osteogenic differentiation medium (ODM) and simultaneously treated with 500 nM NF-κB inhibitor ACHP for 3 days. Gene expression of DLX3 (A), RUNX2 (B) and COL1A2 (C) was measured by RT-qPCRs. Bar charts show means + SD (n = 3). DLX3 Distal-less homeobox 3, RUNX2 Runt-related transcription factor 2, COL1A2 Collagen type I alpha 2 chain. Student’s t-test was performed to compare treatment and control group, but no significant difference was detected. [file 13287_2021_2313_MOESM5_ESM.tif]

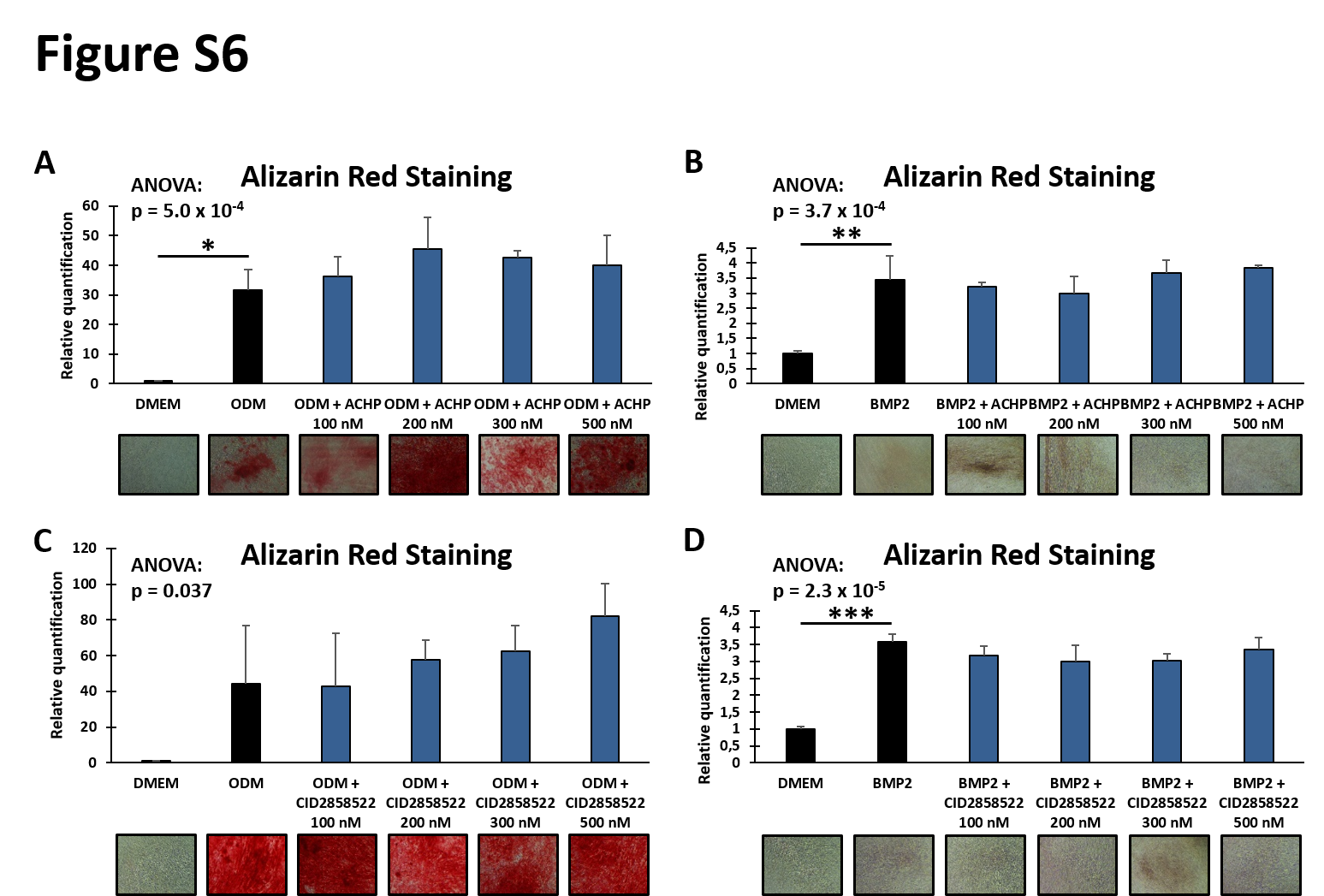

Supplement: Supplementary file 6 — Additional file 6: Figure S6. Mineralization after NF-κB inhibition. DFCs were cultivated in osteogenic differentiation medium (ODM, A, C) or BMP2 containing differentiation medium (B, D) and simultaneously treated with different concentrations of NF-κB inhibitors ACHP (A, B) or CID2858522 (C, D) or cultivated in control medium (DMEM) for 28 days before mineralization was determined by Alizarin Red staining (total width of each photograph corresponds to 1.24 mm). Bar charts show means + SD (n = 3). One-way ANOVA was performed to compare all groups including Tukey’s post hoc tests comparing different groups in the same medium pairwise or DMEM to ODM/BMP2 control group. *p < 0.05, **p < 0.01, ***p < 0.001. [file 13287_2021_2313_MOESM6_ESM.tif]
